# Myeloid Malignancies Beyond the Cell: Targeting the Tumour Microenvironment with Next-Generation Immunotherapies

**DOI:** 10.3390/cancers18111808

**Published:** 2026-06-01

**Authors:** Niloofar Amirian, Anya Squires, Lauretta Azanabor, Claire L. Walker, Matthew J. Simmonds, Ciro Rinaldi

**Affiliations:** 1 School of Natural Sciences, College of Health & Science, University of Lincoln, Brayford Pool, Lincoln LN6 7TS, UK; namirian@lincoln.ac.uk (N.A.); clwalker@lincoln.ac.uk (C.L.W.); msimmonds@lincoln.ac.uk (M.J.S.); 2 United Lincolnshire Teaching Hospitals, NHS Trust, Lincoln LN2 5QY, UK

**Keywords:** myeloid malignancies, myeloproliferative neoplasm, myelodysplastic syndromes, immunotherapy, tumour microenvironment

## Abstract

Myeloid malignancies encompass a heterogeneous group of haematological disorders, primarily including myelodysplastic syndromes (MDSs) and myeloproliferative neoplasms (MPNs). MDS is characterised by defective myeloid cell maturation, while MPNs involve the pathological overproduction of myeloid lineage cells. In the absence of timely diagnosis and effective clinical intervention, both entities carry a substantial risk of progression to acute myeloid leukaemia (AML). Although allogeneic haematopoietic stem cell transplantation remains the only potentially curative therapy, its application is frequently constrained by patient-related factors such as advanced age and comorbid conditions. While currently, hypomethylating agent therapy (azacitidine and decitabine) is mainly used in high-risk MDS patients, and ruxolitinib is primarily used in symptomatic primary myelofibrosis (PMF-MPN), their clinical efficacy remains suboptimal. More recently, focus has turned toward the role of the tumour microenvironment (TME) in disease pathogenesis and whether therapeutically targeting the TME, either alone or in combination with conventional therapy, could present a new treatment option. Emerging evidence underscores the significant influence of TME components, particularly macrophages and T cells, in modulating immune responses and shaping the leukaemic niche to either facilitate or hinder malignant progression. In response, a new generation of immune checkpoint inhibitors are being developed to target the TME, including PD-1/CTLA-4 blockers, macrophage-directed agents including CD47 inhibitors, and T cell-targeting checkpoint inhibitors such as TIM-1 and LAG-3. This review will describe the functional role of key TME constituents in the progression of myeloid malignancies and explore the current landscape and future potential of advanced cellular and molecular immunotherapies in the treatment of these disorders.

## Simple Summary

Myelodysplastic syndrome and myeloproliferative neoplasms are a group of conditions characterised by alterations in myeloid cell production and can evolve into acute myeloid leukaemia (AML). While bone marrow transplantation is the only curative option, it is not applicable for all patients due to their age and the presence of secondary complications. Other therapeutic approaches such as hypomethylating agents or pathway inhibitors are only effective in around half of patients, suggesting an urgent need for improved therapeutic strategies that halt disease progression and reduce the risk of leukaemic transformation. More recently, interest in immunotherapy, specifically approaches that can modulate immune surveillance to promote an anti-tumour microenvironment, has significantly grown. In this review, we discuss emerging immune checkpoint targets and a range of immunotherapeutic inhibitors targeting molecules such as CD47. Our review also summarises recent developments in the field, ongoing challenges, and outlines potential future research directions needed to bring these new approaches fully into practice.

## 1. Introduction

### 1.1. Challenges in Treatment of Myeloid Malignancies

Myeloid malignancies are a group of clonal disorders characterised by either uncontrolled proliferation or impaired differentiation of pluripotential haematopoietic stem cells (HSCs) and often progress to acute myeloid leukaemia (AML). In Europe, these conditions affect approximately 8.6 individuals/100,000 people each year [1,2]. These disorders are broadly classified into three main categories: myeloproliferative neoplasms (MPNs) [3]; myelodysplastic syndromes (MDSs), characterised by defective HSC differentiation and abnormal cellular morphology [4]; and finally, AML, an acute transformation defined by the accumulation of immature malignant blast cells [5].

MDSs are a diverse group of conditions with a range of different genetic and epigenetic markers and are categorised by increased presence of blasts, dysplastic haematopoiesis, and dysplastic megakaryopoiesis [6,7]. MPNs, on the other hand, are primarily associated with mutations in Janus kinase 2 (JAK2V617F), which leads to continuous activation of the JAK-STAT signalling pathway in leukaemic stem cells and over-proliferation of differentiated myeloid cells [8,9]. MPNs are typically divided into three subtypes: Essential Thrombocythemia (ET), Polycythemia Vera (PV), and Primary Myelofibrosis (PMF). They can be identified by either persistent cytosis, including erythrocytosis in PV and thrombocytosis in ET, or advanced fibrosis, cytopenias and abnormal megakaryocyte maturation in PMF. In contrast, AML is defined as both an independent disease and a secondary condition into which MDS and MPNs develop. In secondary AML, mutations in genes such as Additional sex combs-like protein 1 (ASXL1) and Tet methyl cytosine dioxygenase 2 (TET2), involved in regulating DNA methylation, have been associated with worse prognoses and reduced survival rates [10,11]. In contrast, de novo AML typically presents with somatic mutations in FMS-like receptor tyrosine kinase 3 (FLT3) and nucleophosmin 1 (NPM1), which tend to respond more effectively to conventional AML therapies compared to AML arising from MDS and MPN (fully reviewed in [12,13,14,15]).

Bone marrow blasts, the number of cytopenias, and cytogenetics form a key part of the International Prognostic Scoring System (IPSS) and the Molecular IPSS (IPSS-M) scores used to evaluate MDS clinical prognosis, survival rates, and therapeutic approaches [16,17], while also classifying patients into low-, intermediate-, and high-risk groups. Similarly, the diagnosis of MPN relies on genetic assessment in combination with clinical criteria, haematologic measurements and cellular morphology [9]. The complex pathophysiology of chronic myeloproliferative disorders has made it challenging to establish a universal treatment approach for all patient groups. Although allogeneic haematopoietic stem cell transplantation (HSCT), as the only curative option, has shown encouraging results, including a 64.4% two-year overall survival in MPN patients [18], and a 39% two-year survival rate in MDS patients [19], its application is limited due to factors like patients’ age, the presence of comorbidities, and metastatic solid tumours present in 54% of ineligible MDS cases [20,21]. Thus, treatment strategies for these patients have largely centred on chemotherapy to relieve symptoms or prevent further progression in low-risk groups, progressing to use of more targeted agents, such as azacitidine and decitabine for MDS, and ruxolitinib for MPN in higher-risk cases.

While hypomethylating agents (HMAs), including azacytidine (AZA) and decitabine, are the mainstay treatment approach for MDS patients, studies have revealed that overall survival and clinical outcomes in MDS and MPN patients with accelerated blast phase treated with AZA stand at only approximately 24.5 and 6.7 months respectively [22,23]. With a lack of new therapies being developed for MDS and MPN over the last twenty years, more recently, therapeutic strategies have turned to using novel combination strategies, which also focus on targeting the tumour microenvironment (TME), to enhance HMA-based treatments [24,25]. In this review, we aim to critically evaluate the impact of components of the TME on MDS and MPN, pathogenesis with AML discussed mainly as a transformation endpoint, and to discuss recent innovations and challenges encountered when using immunotherapeutic strategies to treat these conditions.

### 1.2. Impact of Metabolism and Epigenetic Regulation of the TME on Myeloid Disorders

Accumulating evidence indicates that both the innate and adaptive immune system become aberrantly activated in MDS and MPN patients as the malignant clone expands, leading to a deleterious state of chronic inflammation in the bone marrow environment. It has been established that rapid cell division, uncontrollable cancer cell growth, and prolonged survival in the competitive bone marrow environment require unlimited fuel support. To meet these demands, cancer cells are capable of hacking metabolic pathways and nutrient acquisition, resulting in sustained cell growth and proliferation. A growing body of research has increasingly highlighted the importance of metabolic reprogramming in cancer progression [26].

In MDS, recent studies have shown that malignant cells create a highly oxidative environment resulting from a dysregulated metabolic process and mitochondrial dysfunction. Notably, these leukaemic clones preferentially use aerobic glycolysis, known as the Warburg effect, over oxidative phosphorylation (OXPHOS), and fatty acid oxidation (FAO), leading to massive reactive oxygen species (ROS) production as a byproduct of mitochondrial metabolism [27,28]. This oxidative stress further contributes to DNA damage, lipid peroxidation, protein oxidation, and, more importantly, overexpression of hypoxia-inducible factor 1-alpha (HIF-1α). As a key transcriptional regulator, HIF-1α upregulates the expression of genes involved in angiogenesis and cell proliferation, including c-MYC, vascular endothelial growth factor (VEGF), and epidermal growth factor (EGF) [29], ultimately driving disease progression and leukaemic cell survival. Additionally, somatic mutations affecting metabolic regulators such as isocitrate dehydrogenase (IDH), reported in approximately 5–12% of MDS patients, play an essential role in metabolic perturbation, leading to further epigenetic mutations in MDS [30].

Similarly, in MPN, the most common driver mutation, JAK2V617, disturbs metabolic homeostasis in the TME by increasing the expression of nutrient transporters such as Glut 1 and glycolytic enzymes including 6-phosphofructo-2-kinase/fructose-2,6-bisphosphatase 3 (PFKFB3) [31]. This upregulation enhances glucose uptake and accelerates glycolysis, thereby providing a favourable growth condition for malignant cells. Moreover, epigenetic studies have recently revealed that additional mutations, such as Enhancer of Zeste Homolog 2 (EZH2) and histone deacetylases (HDAC), significantly contribute to metabolic reprogramming in malignant cells. This may explain the inefficacy of JAK2 inhibitors in certain MPN patients who carry these epigenetic alterations [32,33].

Whilst systemic metabolic alterations are frequently reported in the initiation and progression of myeloid malignancies, these changes not only assist mutated malignant haematopoietic stem cells in evading immune surveillance but also lead to modifications in the TME that further support tumour progression.

In general, in low-risk MDS, the bone marrow environment is dominated by pyroptosis, which is a form of inflammatory cell death, and intramedullary apoptosis. These events, together with elevated cytokine levels, create a highly inflamed bone marrow niche. However, in high-risk MDS and AML, the cellular dysfunction and severely exhausted normal HSCs create an extremely suppressed environment, where hyperactivated T regulatory (Treg) cells and multiple immune checkpoint molecules enable malignant cells to evade immune surveillance [25,34].

Studies have further revealed that disease-initiating malignant haematopoietic stem cells, known as leukaemic stem cells (LSCs), have key mutations in genes encoding epigenetic modifiers, such as TET2, ASXL1, DNA methyltransferase 3A (DNMT3A), EZH2, and IDH1/2 [35]. These mutations disrupt normal DNA methylation and histone modification patterns, leading to aberrant gene expression not only in LSCs but also in nearby stromal and immune cells. Such changes play a major role in the pathogenesis of myeloid disorders, promote treatment resistance and facilitate progression toward AML [36].

Genetic and epigenetic alterations in myeloid malignancies make LSCs difficult to eliminate and trigger progeny expansion. This occurs either by directly increasing pro-inflammatory cytokine production, as in the case of TET2 mutations, which are associated with elevated TNF-α and IL-1β levels, or more directly by creating an inflammatory milieu driven by faulty spliceosome machinery, impaired DNA repair, and dysregulated transcription and signalling pathways. Together, these processes not only support the survival and growth of malignant clones but also ultimately contribute to constant innate immune responses and the activation of the NRP3-inflammasome [37,38].

In low-risk MDS specifically, studies have shown that LSCs acquire the ability for NRP3-inflammasome assembly through ROS and S100A9 production, triggering inflammasome-mediated pyroptosis, which leads to cytopenias that have become a hallmark of the disease [39]. Therefore, a stable inflammatory TME contributes to further genetic instability and accelerated malignant stem cell proliferation, resulting in immune dysregulation and escape from immune surveillance.

Together, these alterations shift the bone marrow milieu into a suppressed and immunologically tolerant environment by activating immune checkpoints, ultimately supporting disease progression. To better understand how myeloid clones alter and adapt the TME to support tumour progression in MDS and MPN, we will discuss major contributing factors to TME disruption, including cytokine aberrations, immune cell dysfunction, and stromal cell-mediated effects, and consider how targeting them may enhance disease treatment.

### 1.3. Alterations in Cytokine Milieu

Cytokines are central mediators in cell–cell communication, facilitating normal haematopoiesis and regulating inflammation/immune responses. However, in myeloid malignancies, cytokine profiles are significantly altered [40,41]. Despite reported discrepancies in cytokine patterns, mostly associated with their dynamic nature and differences in the protocols used to collect patients’ plasma samples, a meta-analysis of 11 studies containing 697 individuals showed that tumour necrosis factor-alpha (TNF-α), interleukin-6 (IL-6), and interleukin-8 (IL-8) were markedly elevated in MDS patients [41], reflecting a pro-inflammatory profile in the bone marrow niche.

TNF-α, a pro-apoptotic cytokine, plays a disruptive role in HSC survival by driving them out of quiescence, resulting in reduced HSC self-renewal capacity and impairing their commitment to the generation of early lineage progenitors. This ultimately results in an extremely exhausted stem cell population sensitive to DNA damage due to increased proliferative pressure. However, this detrimental effect can be reversed by removing TNF-α from the surrounding environment, allowing HSCs to regain quiescence features, even after prolonged TNF-α exposure [42]. Therefore, inhibiting TNF-α has emerged as a new target in treatment approaches for myeloid malignancies.

IL-8, a pro-inflammatory cytokine produced by myeloid cells, facilitates crosstalk between HSCs and mesenchymal stem cells (MSCs), while also regulating myeloid differentiation. In myeloid malignancies, upregulated IL-8 levels arise from myeloid malignant clones and activate signalling pathways such as MAPK, NF-κB and JAK-STAT, leading to the creation of an inflammatory TME that ultimately supports malignant haematopoiesis. Studies have revealed that elevated levels of IL-8 are highly associated with bone marrow fibrosis in PMF-MPN patients [43]. In addition, IL-6, as a pleiotropic inflammatory cytokine, regulates innate immune responses, specifically through the recruitment of neutrophils to the site of inflammation and regulation of haematopoiesis. The role of IL-6 across multiple cancer types is well established. However, despite elevated expression of IL-6 in myeloid malignancies, its exact role in disease pathogenesis and immune dysregulation remains poorly defined. Recent investigations in solid malignancies have revealed that IL-6 contributes to tumour progression by activating the JAK2-STAT3 signalling pathway, which promotes cell proliferation, modulates gene expression and ultimately promotes tumour growth [44,45]. Therefore, further research is required to discover its potential as a novel target, and specifically how advanced immunotherapies may offer new opportunities to alter these different pathways to delay tumour progression and inhibit oncogenic signalling.

Additionally, IL-17, an immunomodulatory cytokine that promotes immune suppression through recruiting mesenchymal stem cells [46], is significantly reduced in high-risk MDS patients compared to controls [41], suggesting impaired immune surveillance in high-risk individuals, which may contribute to an unchecked expansion of malignant clones. In contrast, emerging evidence on solid tumours shows a different pattern, with overexpression of IL-17 in cancer stem cell that promotes tumorigenesis, therapeutic resistance and relapse. Elevated levels of IL-17 support cancer stem cell maintenance by inducing a cancer-related gene expression profile, resulting in the upregulation of transcription factors including c-MYC and activated STAT3, ultimately leading to tumour proliferation and invasion [47,48]. Despite these insights, the exact role of IL-17 is not fully elucidated in myeloid malignancies; therefore, further investigation is required to understand the underlying mechanism.

Overall, while specific cytokines have been identified as playing roles in the pathogenesis of MDS and MPN, it is increasingly recognised that broader disruptions in the cytokine environment contribute to disease progression. Advances in analytical techniques are now enabling more accurate and comprehensive profiling of these cytokine changes as the disease evolves. Approaches such as cytokine arrays and other high-throughput platforms allow for improved detection of complex cytokine signatures over time, supporting a more nuanced understanding of disease mechanisms and progression.

### 1.4. Immune Cell Dysfunction

Cellular immunity in MDS/MPN is primarily controlled by CD8+ cytotoxic T lymphocytes (CTLs), which eliminate pathogens or cancer cells by either direct attack or by producing cytokines [49]. However, in high-risk MDS patients, CTLs fail to effectively deliver the “lethal hit” signal to cancer cells. This failure occurs because in MDS, CTLs lose their ability to adhere to cancer cells, which is associated with lower expression of adhesion molecules, including CD11a, CD18 and intercellular adhesion molecule (ICAM), resulting in CTLs’ inability to form an immune synapse needed to support their activation [50].

Treg cells support immune haemostasis by preventing excessive immune responses through the production of anti-inflammatory cytokines, including IL-10 and TGF-β [51]. However, in myeloid malignancies, this suppression plays an important role in protecting malignant stem cells from immune responses, resulting in subsequent disease progression. In high-risk MDS patients, the number of Treg cells increases significantly compared to low-risk patients, correlating with higher blast counts and IPSS scores [52,53]. Despite AZA therapy showing a notable reduction in the overall number of Treg cells, it also upregulates the production of IL-17 by the remaining Treg cells [54]. Because IL-17 triggers aberrant transcription factor expression in LSCs, this shift may explain why some patients develop resistance to HMA therapy.

Tumour-associated macrophages (TAMs), which perform a range of functions including phagocytosis and tissue regeneration, have also been implicated in the development of MDS and MPN [55]. Macrophages, the main phagocytic cells in the bone marrow niche, are a double-edged sword, exhibiting both tumoricidal and pro-tumour features [56]. Activated macrophages are plastic and can be broadly divided into 2 major subgroups: classical M1-like cells, which produce inflammatory cytokines (IL-6, IL-12) triggering T-helper 1 (Th1) immune responses and enhancing phagocytic activity, and alternative M2-like cells, which demonstrate non-inflammatory phenotypes that initiate pro-tumour activities through suppressive cytokines, resulting in T-helper 2 (Th2) responses [57,58,59].

Although TAMs are key elements of the TME, their roles in normal and malignant haematopoiesis remain unclear. In MPN-ET patients, anti-inflammatory M2 macrophages support disease progression by promoting abnormal megakaryocyte maturation and platelet production via PI3K-AKT activation and TGF-β secretion; conversely, pro-inflammatory M1 macrophages inhibit megakaryocyte development [60]. Primary evaluation of macrophage populations in MDS patients identified higher levels of M2 macrophages in high-risk patients, implying that as MDS progresses, macrophages polarise to a more pro-tumour/anti-inflammatory phenotype [61,62]. Supporting this, Xing et al., in a co-culture system using M2 macrophages from high-risk MDS patients, showed that these cells supported LSC survival over normal HSCs [63]. Moreover, work by Zhang et al. demonstrated decreased expression of pro-inflammatory IL-1β and TNF-α mRNA in M1 macrophages in high-risk MDS patients, which attenuates the normal anti-tumour activity of these macrophages, potentially resulting in an exacerbated disease presentation [61]. Therefore, there is significant evidence of macrophage polarisation to an anti-inflammatory/pro-tumour M2 phenotype as a contributing factor in MDS and MPN disease.

### 1.5. Stromal Cells

Myeloid-derived suppressor cells (MDSCs) predominantly arise under pathological conditions such as chronic infections or stress-related states like cancer and exert potent immunosuppressive effects on T cells, macrophages and dendritic cells. Although the exact immunosuppressive mechanism of MDSCs differs according to cancer type, disease stage and MDSC phenotype, their core strategies include depleting essential amino acids, producing nitric oxide (NO) and ROS, and inducing T cell exhaustion by upregulating PD-L1 [64,65].

Various clinical experiments have identified MDSCs as predictive markers in cancers, including solid tumours and haematological malignancies [66]. Kittang et al. showed that MDSCs are markedly elevated in high-risk MDS patients compared to low-risk patients and healthy donors. Moreover, a positive correlation between the percentage of MDSCs and Treg cells was reported in high-risk MDS patients [67], with elevated numbers of MDSCs also seen in MPN patients [68], indicating a highly suppressed immune surveillance in the bone marrow niche, which could potentially drive disease exacerbation and progression to secondary AML.

As our understanding of the TME continues to evolve, it is becoming increasingly clear that TME dysregulation in MDS and MPN is not driven simply by elevated levels of individual cytokines or imbalances within a single immune subset. Instead, disease progression is sustained through coordinated cell-cell communication and ligand-receptor crosstalk between leukaemic stem cells and the surrounding microenvironment. A deeper understanding of how key signalling pathways such as CD47/SIRPα, PD-1/PD-L1, TNF-and α/TNF-receptor, alongside a broader network of cytokine and chemokine interactions, collectively shape an immunosuppressive, pro-tumour niche, is critical. Together, these pathways converge to create a TME that is both immune tolerant and functionally suppressive, supporting leukaemic cell survival while simultaneously disrupting normal haematopoiesis.

Recent technological advances in single-cell transcriptomics, high-dimensional flow cytometry-based phenotyping, single-cell RNA sequencing, and spatial profiling technologies are now enabling a more refined dissection of immune and stromal heterogeneity within the TME [69,70]. These approaches have revealed distinct inflammatory, immunosuppressive, and fibrotic niche states in patients with MDS and MPN, providing mechanistic insight into how malignant clones remodel their microenvironment. Importantly, this reshapes our view of the TME as a spatially organised ecosystem that differs between anatomical niches and disease states. Defining patient-specific microenvironmental signatures will offer opportunities to model TME interactions using advanced in vivo and ex vivo systems to better stratify which immunotherapies and combination therapies with standard therapies may best support the treatment of different patient groups or individuals.

## 2. Immune Checkpoint Inhibitors: Progress and Challenges

While immune system alterations seen in MDS/MPN have created significant challenges to treatment success, our increased understanding of the role of the TME in these conditions holds considerable promise for advancing treatment options. A range of immunotherapies are being explored to target these various pathways. These options fall into three specific areas, including newly emerging approaches such as T- and NK-cell therapies, as reviewed in more detail elsewhere [71,72], and a range of immune checkpoint inhibitors/immunoblockade therapies.

**Figure 1:**
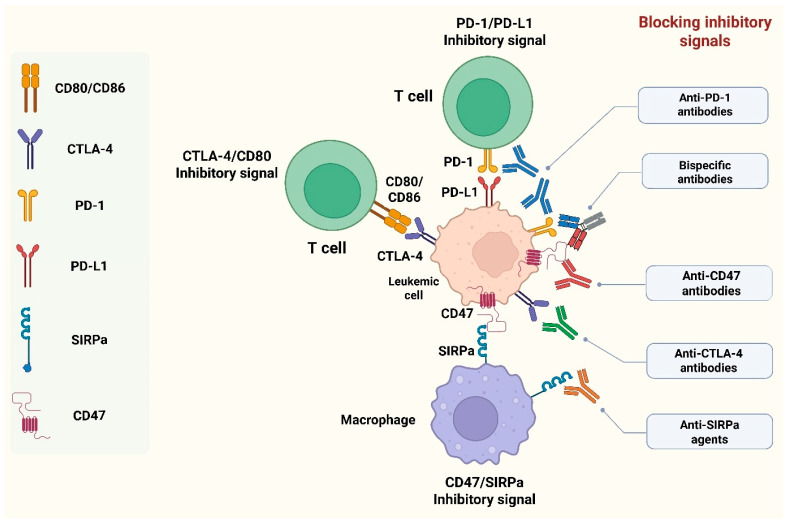
Immune checkpoint inhibitors: Immune checkpoint (ICP) molecules play a critical role in regulating immune responses. Among them, PD-1/PD-L1 and CD47/SIRPα are key checkpoints that modulate anti-cancer immunity. In the PD-1/PD-L1 pathway, PD-1, expressed on T cells, binds to PD-L1 on tumour cells, thereby transmitting an inhibitory signal that suppresses T cell activation and allows tumour immune evasion. Similarly, in the CD47/SIRPα pathway, CD47, expressed on malignant cells, interacts with SIRPα on macrophages, delivering a “don’t eat me” signal that inhibits macrophage-mediated phagocytosis. Inhibition of these pathways with targeted agents can enhance immune-mediated tumour clearance. PD-L1 and CD47, which are expressed on malignant cells, can be blocked using monoclonal antibodies or bispecific antibodies. Likewise, SIRPα, expressed on macrophages as the CD47 ligand, can be inhibited by various therapeutic agents, including monoclonal antibodies and fusion proteins.

Immune checkpoint (ICP) molecules regulate immune responses through ligand-receptor interactions that either inhibit or stimulate downstream signalling pathways. Inhibitory checkpoints in particular act as “brakes” to maintain immune system homeostasis. Blocking inhibitory checkpoints has shown great promise in cancer immunotherapy by enhancing the body’s own anti-tumour responses; however, some concerns remain regarding treatment specificity and the identification of appropriate target populations. Recent advances in targeting well-known inhibitory checkpoints, such as cytotoxic T lymphocyte-associated antigen-4 (CTLA-4) and programmed cell death protein 1 (PD-1), have opened the door to targeting additional immune checkpoints, including CD47, lymphocyte-activation gene 3 (LAG-3), and T cell immunoglobulin and mucin domain 3 (TIM-3) (as shown in Figure 1) [73,74,75]. In this review, we explore the current landscape of immune checkpoint inhibitors and discuss the challenges that must be resolved to unlock their full therapeutic potential.

## 3. PD-1 and CTLA-4 in Myeloid Malignancies

PD-1 and CTLA-4, both expressed on activated T cells, ameliorate immune responses following chronic exposure to stimulants, enhancing self-tolerance [76]. The interaction of PD-1 with its ligands, PD-L1 and PD-L2, initiates intrinsic signalling pathways that recruit tyrosine phosphatase, ultimately reducing antigen presentation to T cells [77], while the binding of CTLA-4 to its receptors, CD80 and CD86, blocks co-stimulatory signals required for T cell activation [78]. The correlation between PD-1 upregulation and poor prognosis has been confirmed in solid and haematological malignancies, indicating the role of elevated PD-1 levels in leukaemic immune evasion through increasing Treg cell activation and inhibition of T cells [79,80].

MDS patients show an upregulation of PD-1, PD-L1, PD-L2, together with CTLA-4 on CD34+ myeloid stem cells, which has been associated with worsening prognosis after treatment with decitabine [81,82]. Ørskov et al. demonstrated that PD-1 promoter methylation was lost in the T cell population of 44% of MDS patients treated with AZA. This indicates that HMA therapies could exacerbate immune exhaustion through hypomethylating the PD-1 promoter, leading to increased PD-1 marker expression on T cells [83]. Immunohistochemistry revealed that MDS blasts mainly express PD-L1, while stromal and non-leukaemic cells are largely PD-1 positive, suggesting a crosstalk between MDS cells and the TME that contributes to immune exhaustion as MDS progresses [82]. Consequently, targeting the PD-1/PD-L1 axis has been considered a potential therapeutic strategy for these patients.

The first FDA-approved anti-PD-1 antibody, Pembrolizumab [84], was initially evaluated as a single-agent therapy in 27 intermediate- and high-risk MDS patients who failed prior treatment with HMA therapy, in the multicentre KEYNOTE-013 (NCT01953692) clinical study. Despite the limited efficacy of the drug, with no complete remission seen and only a single patient achieving partial remission, the study proved that Pembrolizumab is a well-tolerated drug among patients, with only 36% of patients experiencing treatment-related adverse events, including hypothyroidism (14%) and fatigue (11%) [85]. Future studies added in a frontline group with no previous exposure to HMA treatment and a combination strategy with HMA-based therapy to see if this enhanced Pembrolizumab’s ability to trigger patient remission. The phase II clinical trial, performed at the University of Texas MD Anderson Cancer Centre (NCT03094637), enrolled intermediate- and high-risk MDS patients, with 20 patients in the HMA-failure cohort and 10 patients forming a treatment-naïve group, treated with a combination of AZA and Pembrolizumab. The patients were followed up for approximately 13 months and showed an enhanced overall response rate (ORR) of 25% in the HMA-failure group and 76% in the non-treated patients. Despite no significant difference in overall patient survival, remaining at 5.9 months in the HMA-failure group and 12.9 months in the HMA-naïve group, the high-risk MDS patients showed evidence of treatment-related improvement; one patient received the therapy for 31 consecutive months and remained in complete remission (CR) without notable toxicity [86]. These encouraging findings are consistent with additional trials [87], suggesting that the combination of HMA-based therapy with PD-1/PD-L1 blockers represents a novel immunotherapeutic approach in high-risk MDS patients.

Nivolumab, another anti-PD-1 antibody first approved for patients with advanced squamous-cell non–small-cell lung cancer in 2014 [88], was also investigated in newly diagnosed AML and high-risk MDS patients who also received a standard induction regimen (Idarubicin and Cytarabine). Evaluation of responses among 44 enrolled patients showed that 34 (77%) achieved complete response, and of these 34 patients, 18 (53%) had undetectable minimal residual disease following induction therapy. This suggests that the addition of Nivolumab to induction therapy aided in reconstituting effector T cells against leukaemic cells. The findings also showed significantly fewer PD-1^+^ CD4^+^ T-effector cells in responders than in non-responders [89], highlighting the PD-1/PD-L1 pathway’s role in reducing immune system exhaustion, enhancing T cell function and improving treatment efficacy.

More trials have also explored the inhibition of both PD-1 and CTLA-4, in combination with HMA-based regimens. In Guillermo’s 2016 trial, high-risk MDS patients were enrolled either as treatment-naive or those who failed to respond to prior HMA therapy. Participants in both groups received Nivolumab (anti-PD-1) or Ipilimumab (anti-CTLA-4) as monotherapy, or the combination of both agents. The HMA-naive group additionally received AZA to evaluate the benefit of combining immune checkpoint inhibitors with HMA-based therapies. The results demonstrated a 69% ORR in the AZA + Nivolumab cohort compared to only 0% and 22% in the Nivolumab and Ipilimumab monotherapy arms, respectively [90]. These findings highlight the potential of combining immune checkpoint inhibitors with standard HMA therapies, particularly for untreated high-risk MDS patients. A more recent study (NCT02530463) [91], in which both the frontline and HMA-failure groups received the combination of Nivolumab and Ipilimumab with AZA added in the HMA-naive group, showed that ORR was 67% and 36% in the HMA-naive and HMA-failure groups, respectively. This suggests that dual immune checkpoint blockade may offer potential clinical efficacy in both settings. Additionally, immunophenotypic analysis from Guillermo’s trial showed that the expression of PD-1 on the progenitors and malignant stem cell population increased, highlighting that the immunoblockade approach may not reverse immune exhaustion; therefore, novel approaches that are capable of targeting these aberrant exhaustion markers are urgently needed.

## 4. Targeting CD47-SIRPα Axis to Enhance Macrophage-Mediated Phagocytosis

**Figure 2:**
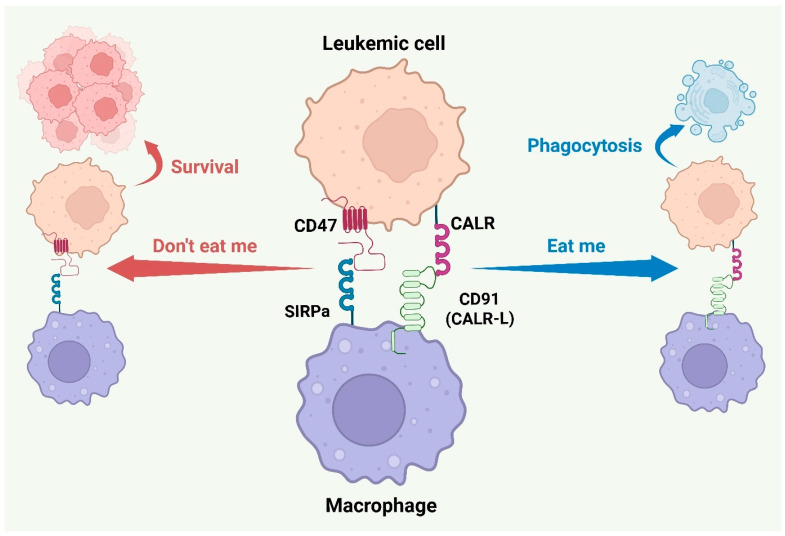
The role of the CD47-CALR axis in the pathogenesis of malignancies. In the TME, the interaction between CD47 on leukaemic cells and its receptor, SIRPα, on macrophages delivers a “don’t eat me” signal, which suppresses macrophage activation and promotes leukaemic cell survival. In contrast, the binding of CALR on cancer cells to its receptor, CD91, on macrophages triggers an “eat me” signal, initiating macrophage activation and subsequent phagocytosis of the malignant cells. SIRPα, signal regulatory protein alpha; CALR, calreticulin; CALR-L, calreticulin ligand.

Recently, CD47 has gained attention as a therapeutic target in both solid tumours and haematologic malignancies. CD47, known as a “don’t eat me” signal, is a transmembrane protein belonging to the immunoglobulin (Ig) superfamily [92], which, upon binding to its receptor, signal regulatory protein alpha (SIRPα), on macrophages, inhibits phagocytosis, enabling cancer cells to evade immune surveillance (Figure 2) [93]. Numerous studies have shown that the overexpression of CD47 on cancer cells is associated with poor clinical outcomes and patient survival [94,95,96].

CD47 is counterbalanced by calreticulin (CALR), a pro-phagocytic signal [97]. CALR is an endoplasmic reticulum (ER)-resident protein involved in various cellular processes, including calcium control, adhesion and integrin signalling [98,99]. In cancer and apoptotic cells, ER stress activates a downstream cascade, leading to CALR translocation from the ER to the cell surface, which facilitates its interaction with CD91 on macrophages, promoting programmed cell removal and ultimately supporting anti-cancer immunity [100] (Figure 2).

Analyses of CD47 and CALR expression in MDS have shown that elevated CD47 correlates with greater disease severity, supporting its potential use as a biomarker for identifying patients likely to benefit from anti-CD47 therapy [101]. In MPN, cytoreductive therapy led to a decrease in CALR expression, while CD47 expression was upregulated [96]. Additionally, in vitro laboratory research suggested that blocking CD47 facilitates the programmed cell removal of cancer cells due to the higher CALR expression [101,102,103,104]. This approach shows promise in enhancing anti-cancer immunity, and as a result, various CD47-blocking strategies are currently being developed and trialled to enhance MDS/MPN treatment.

The first approach involved monoclonal antibody blockade of CD47. Magrolimab (Hu5F9-G4) is an IgG4 monoclonal antibody that blocks the CD47-SIRPα pathway [105], enhancing macrophage-mediated phagocytosis and reducing tumour burden [106]. Although anti-CD47 activity has been demonstrated in acute lymphoblastic leukaemia and non-Hodgkin lymphoma [94,107], magrolimab has not yet received FDA approval.

The initial Phase I clinical trial in myeloid malignancies was conducted by Sallman et al. in 2019 [108] (NCT03248479), enrolling 10 patients with relapsed/refractory AML/high-risk MDS and 24 untreated patients. The preliminary data showed that 53% of previously untreated AML/MDS patients treated with the combination of magrolimab and AZA achieved complete remission (CR). In contrast, only 10% of the patients in the relapsed/refractory group responded to magrolimab monotherapy, highlighting the limited efficacy of the single-agent treatment in this cohort.

Due to the limited patient population in the initial cohort, the study was expanded into a Phase Ib trial, which showed more encouraging outcomes with the combination of magrolimab and standard AZA therapy. The regimen resulted in 100% objective responses, including 54% complete remission (CR), 39% marrow CR, and 7% haematologic improvement (HI) in untreated MDS patients, while it was effective in only 69% of AML patients. The trial also managed to correlate the efficacy of the treatment approach for a specific subgroup, with patients with tumour protein p53 (TP53) mutations showing an 88% objective response [109,110,111].

However, the Phase III ENHANCE study (NCT04313881), which was evaluating the combination of anti-CD47 blockade magrolimab and AZA in MDS patients, was ultimately discontinued in 2023. Although the full details were not extensively reported, the study was stopped due to futility, with concerns over anaemia, posing potential safety risks to patients, and results suggesting that treatment was unlikely to achieve its primary endpoint [112,113]. This was in contrast to the promising results acquired in the earlier Phase I study, with several explanations having been proposed for the limited clinical efficacy of this therapy. Recently, Rinaldi et al. provided preliminary evidence to suggest that the combination of ruxolitinib and magrolimab could significantly increase CALR membrane expression compared with ruxolitinib or magrolimab alone, signalling a much stronger pro-phagocytic message in myelofibrosis cells compared to controls [114]. These findings indicate that the combination of AZA and magrolimab alone may not achieve the essential efficacy for eliminating LSCs through macrophage-mediated phagocytosis. Thus, further investigation is required to evaluate the therapeutic potential of alternative combination regimens in MDS patients.

The second explanation may relate to the immune composition of high-risk MDS patients, who exhibit a predominance of pro-tumour M2 macrophages and reduced pro-inflammatory M1 macrophages, which may represent a less suitable target group. This is supported by the work of Hassan et al., demonstrating that CD47 knockdown led to a significant increase in CALR expression on cancer cells, but only when co-cultured with human monocyte-derived macrophages. This resulted in substantial apoptosis through an indirect mechanism, reaching 90% in HL-60 cells, an AML model, as well as notable phagocytosis in direct co-culture conditions [115]. This highlights the importance of the presence of M1 macrophages within the TME in enabling effective responses to anti-CD47-related therapies, suggesting that further research is required to identify the appropriate study population with a more favourable immune profile for future CD47 blockade therapies.

In addition, some clinical studies revealed that one of the major challenges in targeting CD47, which is highly expressed on healthy cells like red blood cells (RBCs) [116,117], is the presence of off-target side effects such as anaemia [118]. Thus, the next generation of anti-CD47 antibodies, which can disrupt the CD47-SIRPα axis while ensuring the protection of healthy cells, is now being explored (Table 1).

Gentulizumab, a newly designed anti-CD47 antibody that successfully passed in vivo and in vitro studies on mice and non-human primates, resulted in a higher efficacy in binding to CD47 together with a lower affinity to RBCs compared to magrolimab. This prevented off-target side effects and led to a maximum reduction of 26% in RBCs at the highest dose, with levels returning to normal after 22 days [119]. Despite confirming the anti-tumour effect of gentulizumab in an AML cell line and favourable tolerability in cynomolgus monkeys, the recent clinical trial conducted in China (NCT05263271) for relapsed/refractory AML/MDS patients was terminated, with preliminary results from this work not currently published.

Lemzoparlimab (TJ011133), a novel anti-CD47 antibody engineered to spare RBCs, showed encouraging results in the Phase I clinical trial (NCT04202003) for relapsed/refractory AML patients [120]. The treatment was well tolerated among patients and achieved 84.9% and 82.2% receptor occupancy levels on T and CD33+ cells, respectively, at a dose of 10 mg/kg. Due to the limited sample size of 5 patients, the investigations progressed to a Phase II study in newly diagnosed high-risk MDS patients [121]. This trial enrolled 53 MDS patients who received lemzoparlimab in combination with AZA as the standard therapy. Among 29 patients who received the treatment regimen for more than 4 months, the ORR reached 86.2%, including a 31% complete response (CR) rate, 10% haematologic improvement (HI) alone, and 44.8% marrow CR, indicating the efficacy of combination therapy with AZA. Furthermore, a recent finding about enhancing the anti-tumour activity of the AZA + venetoclax regimen in AML mouse models treated with CD47 inhibitors [122] prompted the initiation of a clinical trial (NCT04912063) [123] in 2022 to investigate the efficacy of lemzoparlimab with venetoclax for untreated AML and high-risk MDS patients. The recruitment for this study has been recently terminated.

Another new anti-CD47 prodrug containing a protease substrate that is inactive in healthy cells has also been recently introduced [124]. This specific design allows the antibody to regain activity only after exposure to protease, which accumulates in the TME, thereby limiting the action of the drug to within the TME. Haemagglutination experiments showed a significant reduction in RBC binding using this therapy, indicating that this approach warrants further study.

Beyond CD47 blockade, inhibiting SIRPα may offer a therapy with fewer side effects due to its significant expression on macrophages [125,126], while still being able to block the “don’t eat me” signal from cancer cells [127]. Despite blocking SIRPα on macrophages and removing the inhibitory signal, macrophages are often not capable of initiating phagocytosis and tumour cell engulfment without an activating signal. Therefore, it highlights the need for novel strategies that remove the inhibitory signals while also providing pro-phagocytic activation.

Recently, bispecific antibodies have also been developed that can simultaneously bind different targets on leukaemic cells. As illustrated in Figure 1, a dual inhibitor, IBI322, which binds to PD-L1 and CD47 on cancer cells, showed enhanced binding to leukaemic cells while attenuating non-specific binding to RBCs [128]. Although the efficacy of these bispecific antibodies has not been evaluated in MDS and MPN disorders [129], these targeted strategies have the potential to improve therapeutic efficacy while reducing the adverse effects commonly linked to CD47-directed antibodies. This suggests that targeting the CD47 pathway could still provide an important step forward in the management of haematologic malignancies, with further refinements needed for this approach to ensure specificity and strong clinical outcomes.

**Table 1:** Different CD47 and SIRPα blockade agents.

Agent	Drug Type	Target	Treatment Plan	Clinical Trials	Indication	Study Population	Study Status (Ref.)	NCT	Outcomes
Magrolimab (Hu5F9-G4)	CD47 mAb	CD47	Magrolimab monotherapy	Phase I	AML	19	Completed [130]	NCT02678338	AEs: Hb declined Increased transfusion requirements Invalid ABO blood typing
Letaplimab (IBI188)	CD47 mAb	CD47	Letaplimab + AZA Letaplimab + Decitabine	Phase I/II	AML	222 (Estimated)	Suspended	NCT04485052	N/A
			Letaplimab + AZA	Phase I	MDS	93	Suspended [131]	NCT04485065	AEs: 49.5% decreased platelet count 44.1% anaemia 36.6% decreased neutrophil count 34.4% haemolysis 82.2% ORR including 31.1% CR 35.6% marrow CR 15.6% HI
Lemzoparlimab (TJ011133)	CD47 mAb	CD47	Lemzoparlimab monotherapy	Phase I	AML	5	Completed [120]	NCT04202003	AEs: thrombocytopenia in 2/5 pts, 1 with grade 1 and 1 with grade 3 positive anti-erythrocyte antibody in 2/5 pts
			Lemzoparlimab + AZA	Phase II	MDS	53	Completed [121]	NCT04202003	AEs: 60% decreased platelet count 53% decreased neutrophil count 40% anaemia 86.2% ORR including 31% CR 10% HI 44% marrow CR
Ligufalimab (AK117)	CD47 mAb	CD47	Ligufalimab + AZA	Phase I	MDS	72	Active, not recruiting [132]	NCT04900350	AEs: 30.6% anaemia 77.8% decreased neutrophil count 72.2% decreased white blood cell count 69.4% decreased platelet count 58.3% pyrexia ORR including 48.1% CR
			Ligufalimab + AZA	Phase I/II	AML	22	Active, not recruiting [133]	NCT04980885	AEs: 32.5% anaemia 70% decreased white blood cell count 55% decreased platelet count 52.2% pyrexia ORR including 55% CR 35% Stable disease
			Ligufalimab + AZA Placebo + AZA	Phase II	MDS	90	Recruiting [134]	NCT06196203	N/A
Urabrelimab (SRF231)	CD47 mAb	CD47	Urabrelimab monotherapy	Phase I	Haematological cancer	46	Completed [135]	NCT03512340	AEs: 2 pts showed febrile neutropenia, haemolysis
CC-90002	CD47 mAb	CD47	CC-90002 monotherapy	Phase I	AML/MDS	24	Terminated [136]	NCT02641002	Serious AEs: 46% diarrhoea 39% thrombocytopenia 36% febrile neutropenia, elevated AST 32% anaemia: Elevated ALT 82% dependent RBC transfusion
Evorpacept (ALX148)	SIRPα/Fc fusion protein antibody	CD47	Evorpacept + AZA	Phase I/II	MDS	13	Active, not recruiting [137]	NCT04417517	AEs: 31% febrile neutropenia 23% pneumonia 23% anaemia 15% thrombocytopenia
			Evorpacept + AZA + Venetoclax	Phase I/II	AML	14	Terminated [138]	NCT04755244	AEs: 43% anaemia 36% elevated AST 29% decreased platelet count 21% pneumonia
Timdarpacept (IMM01)	SIRPα/Fc fusion protein antibody	CD47	Timdarpacept + AZA	Phase I/II	MDS	57	Unknown status [139]	NCT05140811	AEs: 78.9% leukopenia 66.7% thrombocytopenia 66.7% neutropenia 43.9% anaemia 15.8% infection 10.5% pneumonia 64.7% ORR including 29.4% CR, 15.7% marrow CR + HI, 5.9% HI, 13.7% marrow CR
TQB2928	Blocking molecule	CD47/SIRPα	TQB2928 + AZA	Phase I	AML/MDS	48 (Estimated)	Unknown status	NCT06008405	N/A
AUR103 Calcium	Blocking molecule	CD47	AUR103 Calcium	Phase I	AML, MDS, NHL	27 (Estimated)	Recruiting	NCT05607199	N/A

AEs: adverse events, NHL: non-Hodgkin lymphoma, AML: acute myeloid leukaemia, MDS: myelodysplastic syndrome, NCT: National Clinical Trial, Ref: references, ORR: overall response, CR: complete remission, HI: haematological improvement. Clinical trials involving CD47 are listed in the U.S. National Clinical Trial (NCT) registry “https://clinicaltrials.gov/ (accessed on 21 May 2026)". Drug-related terminology was examined using EVS Explore “https://evsexplore.semantics.cancer.gov/evsexplore/welcome (accessed on 21 May 2026)”. All included adverse events were >Grade 3.

## 5. Targeting T Cell Activation Through Tim-3

**Figure 3:**
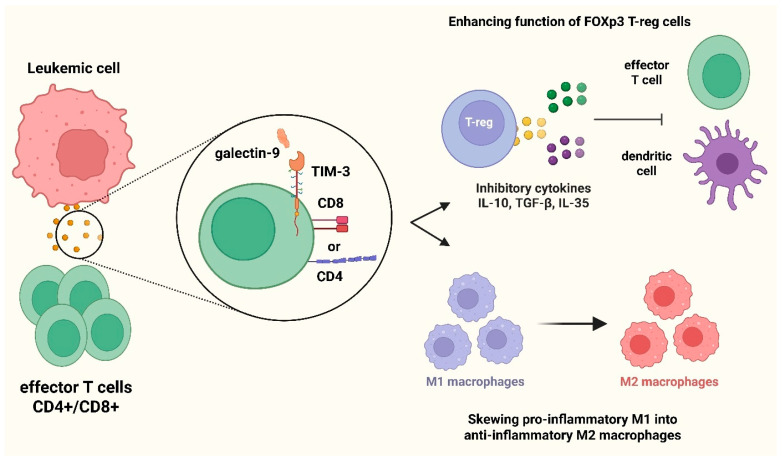
TIM-3 signalling within the TME and its role in immune suppression: TIM-3 is predominantly expressed on T cell populations, including exhausted effector T cells. Leukaemic cells within the TME secrete the soluble ligand galectin-9, which binds to TIM-3 on immune cells, initiating downstream inhibitory signalling pathways. Activation of TIM-3 signalling enhances the immunoregulatory function of Treg cells by inducing the production of inhibitory cytokines, including TGF-β, IL-10 and IL-35, which suppress effector T cells and dendritic cells. In parallel, TIM-3 engagement promotes macrophage polarisation from the pro-inflammatory M1 phenotype to the anti-inflammatory M2 phenotype, further contributing to an immunosuppressive, pro-tumour environment. The TIM-3/galectin-9 interaction therefore represents a key immunological checkpoint and a potential therapeutic target for checkpoint blockade strategies aimed at restoring anti-tumour immune responses.

T cell immunoglobulin and mucin domain 3 (TIM-3), originally expressed on T-helper 1 (Th1) cells, acts as a negative regulator to downregulate immune responses [140]. TIM-3, through its interaction with its ligand galectin-9, induces Th1 cell depletion, thereby preventing autoimmunity and allergic reactions [141]. Conversely, higher expression of TIM-3 on various immune cells, including Treg cells, macrophages and dendritic cells, can help leukaemic cells escape immune responses by promoting T cell exhaustion, enhancing the regulatory function of Treg cells, and skewing pro-inflammatory M1 macrophages to an anti-inflammatory M2 phenotype (Figure 3), ultimately supporting immune suppression and tumour progression [140,142].

Several clinical studies have revealed an increased TIM-3 expression on cytotoxic CD8+ T cells in MDS patients compared to healthy controls. This upregulation of TIM-3 on CD8+ T cells significantly affects the production of IFN-γ, perforin and granzyme B, key pathways in cancer cell destruction, ultimately leading to an impaired CD8+ T cell population [143]. Additionally, the elevated expression of TIM-3/galectin-9 on leukaemic stem cells triggers an autocrine loop that promotes their proliferation [144]. Therefore, blocking the TIM-3/galectin-9 axis to inhibit malignant blast proliferation and improve CD8+ function has been suggested as a therapeutic strategy for MDS.

Ongoing clinical trials are evaluating the potential of the novel anti-TIM-3 antibody sabatolimab (MBG453), in combination with conventional therapies for MDS patients. Sabatolimab is a dual-antagonist antibody that blocks TIM-3 on LSCs, inhibiting autocrine self-renewal while enhancing T cell-mediated anti-tumour responses. A Phase Ib clinical trial (NCT03066648) enrolled 19 high-risk MDS and 50 newly diagnosed or refractory AML patients and assessed the efficacy of sabatolimab with AZA and decitabine, showing promising results. Both combinations were well tolerated, with an overall response rate of 58% in MDS patients treated with sabatolimab + decitabine (5 CR, 4 marrow CR, 2 HI) and 70% in those receiving sabatolimab + AZA (6 marrow CR, 1 PR) [145]. However, because this early-phase trial was conducted in a non-randomised manner and included a very small MDS population, further studies were launched to validate these findings. Zeidan et al. in 2024 enrolled 127 MDS patients in a Phase II randomised multicentre trial (STIMULUS-MDS1, NCT03946670) [146]. In this trial, participants received either sabatolimab + HMA agent or placebo + HMA agent. Preliminary data revealed that the ORR was 22% (14/65 patients) in the sabatolimab arm, while patients treated with placebo showed an 18% ORR (11/62 patients); however, this difference was not statistically significant (*p* = 0.7). Additionally, no significant difference was observed between the two groups in median progression-free survival (11.1 months in the sabatolimab arm versus 8.5 months in the placebo group). Despite the encouraging results in the Phase Ib study, the STIMULUS-MDS1 trial did not confirm the clinical efficacy of sabatolimab in enhancing ORR or survival rates, suggesting that further investigation to elucidate the molecular mechanisms underlying TIM-3 targeted therapies is needed.

In addition to this, co-culture experiments using THP-1-derived macrophages with leukaemic cell lines, including SKM-1, expressing TIM-3, demonstrated that treatment with sabatolimab can enhance macrophage phagocytic uptake through antibody-dependent cellular phagocytosis (ADCP); however, this effect requires the presence of TIM-3 on leukaemic blasts [147]. Therefore, further combination studies incorporating sabatolimab as an anti-TIM-3 antibody alongside other immunotherapy agents or existing treatments are now required to fully realise the utility of targeting TIM-3 in myeloid disorders.

## 6. Lymphocyte Activation Gene-3 (LAG3)

**Figure 4:**
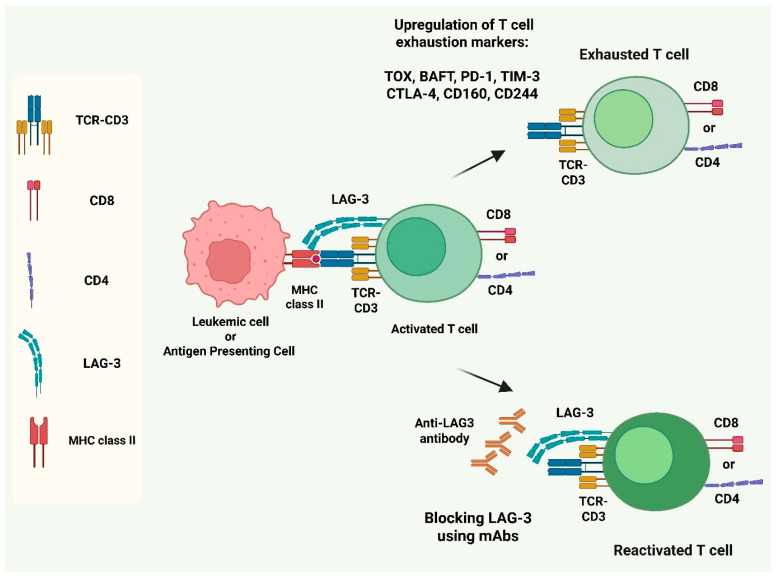
The role of LAG3 in immune suppression: LAG-3 is an inhibitory immune checkpoint receptor expressed on activated T cells. It binds to MHC class II molecules expressed on antigen-presenting cells (APCs), such as dendritic cells and macrophages, as well as on leukaemic cells within the tumour microenvironment (TME). Ligand binding activates the KIEELE motif within the cytoplasmic domain of LAG-3 in T cells, initiating downstream inhibitory signalling pathways. This signalling reduces T cell receptor (TCR) activation, promotes the expression of exhaustion markers, and suppresses effector T cell proliferation and cytokine production. Therapeutic blockade of LAG-3 using monoclonal antibodies can disrupt this inhibitory interaction, restore T cell activation and enhance anti-tumour immune responses. TOX, thymocyte selection-associated high mobility group box protein; BATF, basic leucine zipper ATF-like transcription factor; PD-1, programmed cell death protein 1; TIM-3, T cell immunoglobulin and mucin-domain containing-3; CTLA-4, cytotoxic T-lymphocyte antigen 4.

LAG3 (CD223) is the third checkpoint inhibitor that has garnered significant interest in cancer immunotherapy. LAG-3 is largely expressed on activated NK and T cells to control overactivation [148]. Unlike T cell regulatory molecules such as CTLA-4 and PD-1, LAG-3 does not provide direct inhibition of T cell signalling. Rather, in vitro studies have demonstrated that the interaction of LAG-3 with MHC class II molecules present on malignant cells or antigen-presenting cells can trigger a unique negative regulatory signalling pathway in activated T cells. This leads to the suppression of T cell function, upregulation of T cell exhaustion markers, and ultimately attenuates immune responses against cancers [149,150,151]. Additionally, immunophenotypic studies have demonstrated that blocking LAG-3 on CD8+ T cells with monoclonal antibodies can effectively reinvigorate their cytolytic functions and enhance proliferation (as demonstrated in Figure 4) [152,153,154].

Further research has identified a specific population of Tregs (CD4+CD25+Foxp3+) expressing LAG-3 in patients with melanoma or colorectal cancers. These cells can suppress activated immune cells through IL-10 production, highlighting the impact of LAG-3 on manipulating tumour immune responses [155,156]. Therefore, blocking LAG-3 along with other immune checkpoints has potential as a novel combination therapeutic strategy.

There is no clear-cut evidence regarding LAG-3 expression in MDS and MPN patients. However, Hyun et al. reported that overt PMF-MPN cases display markedly elevated LAG-3 levels on effector T cells compared to pre-fibrotic PMF-MPN patients [157]. Furthermore, a recent study has shown that a higher percentage of CD8+ cells express LAG-3 in MDS patients compared to healthy control counterparts, suggesting a profoundly suppressed TME in MDS patients. Moreover, CD8+ cytotoxic T cells show significantly higher expression of LAG-3 in newly diagnosed MDS patients with TP53 mutations compared to the wild-type group and healthy controls. This finding indicates the potential impact of TP53 mutations on the severity of T cell exhaustion [158], thus suggesting that future investigations should focus on the importance of blocking immune checkpoints in TP53-mutated patients to reprogram exhausted T cells.

In contrast, newly diagnosed AML patients exhibited a significant upregulation of LAG-3 and CTLA-4 expression on T cells. As inhibitory checkpoints, their increased expression was linked to poor prognosis, suggesting their potential use as prognostic markers [159,160]. Moreover, Abdelhakim showed that using an anti-LAG-3 antibody, in a co-culture system with AML cell lines and patient PBMCs, can restore immune system responses by reducing the number of Treg cells and enhancing T cell activation [161]. Recent in vitro experiments on bone marrow aspirates from AML patients showed that LAG-3 and TIM-3 have a strong tendency to be co-expressed with PD-1 on CD8+ T cells, resulting in faster immune exhaustion, which is negatively correlated with overall survival in these patients [162,163]. Therefore, work is needed to assess LAG-3 expression in MDS and MPN patients and its correlation with disease progression into secondary AML to determine if targeting this pathway could support enhancing the immune response in these patients.

## 7. Future Opportunities and Challenges in Immunotherapy

The introduction of immune checkpoint blockade as a novel cancer immunotherapy approach has the potential to lead to improvements in patients’ survival and treatment responses. Unlike standard therapies such as intensive chemotherapy, which aims to eradicate the affected malignant clones, immunotherapy approaches leverage the patient’s own immune system to regulate uncontrolled tumour growth and reinvigorate immune cell function. Immunotherapy strategies, including immune checkpoint inhibitors, cancer vaccines, and CAR-T cell therapies, have shown encouraging outcomes in patients with solid malignancies and certain types of haematological disorders [164]. However, their application remains limited to specific types of cancer, highlighting the importance of further research to expand their usefulness.

Despite promising outcomes in both haematological malignancies and solid tumours, the emergence of immunotherapy resistance has recently posed new challenges associated with this therapeutic strategy. The underlying mechanisms and the impact of therapeutic resistance on disease progression have been well studied in solid tumours [165,166]; however, further investigation that can fully recapitulate the MDS/MPN immune environment is needed in myeloid malignancies, due in part to limitations around representative animal models and advanced 3D culture systems, and to factor in disease heterogenicity.

Immunotherapy resistance is broadly categorised into primary and adaptive resistance, both of which promote disease progression by suppressing immune responses. Primary resistance arises from intrinsic mechanisms in LSCs that fail to respond, while in adaptive resistance, LSCs initially respond to immune checkpoint inhibition but gradually adapt their resistance mechanisms, leading to relapse or leukaemic progression after treatment. Immunotherapy resistance can occur through multiple pathways, including tumour-intrinsic factors, TME-related influences, and host-related factors, all of which ultimately create a highly immunosuppressive environment that favours the malignant cells [167].

Recent studies have indicated that cancer stem cells in solid tumours employ varying strategies to escape immune detection during immunotherapy episodes. These include downregulating tumour-antigen presentation through the loss of β2M as an essential part of MHC class I molecules [168], limiting T cell expansion by introducing novel immune checkpoints such as Siglec-15 [169], and impairing T cell migration resulting from downregulation of CXCL9 and CXCL10, driven by additional somatic mutations involving epigenetic regulators such as DNMTs [170]. In addition, the resistance linked to the TME promotes T cell exhaustion with a highly suppressed environment, characterised by the recruitment of immunosuppressive cells like MDSCs and M2 macrophages, the secretion of anti-inflammatory cytokines, and metabolic reprogramming [165].

Beyond immune system-related mechanisms, host-related factors such as age, gender, obesity and gut microbiome also strongly contribute to immunotherapy resistance and further disease progression [166]. This highlights the importance of identifying resistance-associated biomarkers and developing novel treatment approaches to effectively overcome this challenge. Therefore, patient stratification according to genetic mutations, immune reactiveness, disease stage, reliable response biomarkers and baseline clinical characteristics is strongly suggested to further support the advancement of personalised medicine and to enable the effective use of next-generation immune checkpoint inhibitors along with conventional treatment to provide durable clinical responses in MDS and MPNs.

## 8. Conclusions

Given the rising global prevalence of myeloid disorders, targeting elements of the TME, such as macrophages and T cells, is emerging as a promising strategy to improve treatment outcomes in both MDS and MPNs. Further research is now needed to fully harness the potential of targeting immune checkpoints, both as monotherapy and, particularly regarding their effectiveness, in combination with standard hypomethylating agents/JAK inhibitors that directly target the leukaemic cells as a way of improving clinical outcomes. Additionally, although the mechanisms underlying relapse in AML are well investigated, further research is needed to better understand immunotherapy resistance in myeloid malignancies. While further refinements are needed to determine the most effective ways to target different immune pathways, ensure specificity of therapy, minimise off-target effects, and deepen our understanding of how the TME evolves during disease progression and varies across patients, targeting immune surveillance pathways is providing a new avenue for enhancing treatment outcomes and patient quality of life across a wide range of myeloid neoplasms.

## Data Availability

No new data were created or analysed in this study. Data sharing is not applicable to this article.

## Abbreviations

The following abbreviations are used in this manuscript:

AML	Acute myeloid leukaemia
MDS	Myelodysplastic syndrome
MPN	Myeloproliferative neoplasms
TME	Tumour microenvironment
JAK2	Janus kinase 2
ET	Essential thrombocythemia
PV	Polycythemia vera
PMF	Primary myelofibrosis
ASXL1	Additional sex combs-like protein 1
TET2	Tet methyl cytosine dioxygenase 2
FLT3	FMS-like receptor tyrosine kinase 3
NPM1	Nucleophosmin 1
IPSS	International Prognostic Scoring System
HSCT	Hematopoietic stem cell transplantation
HMA	Hypomethylating agent
AZA	Azacytidine
LSC	Leukemic stem cell
TNF-α	Tumour necrosis factor-alpha
IL	Interleukin
NF-κB	Nuclear factor κ-light-chain-enhancer of activated B cell
CTL	Cytotoxic T Lymphocyte
Treg	T regulatory
Th	T-helper
MDSC	Myeloid-derived suppressor cell
NK	Natural killer
ICP	Immune checkpoint
CTLA-4	T lymphocyte-associated antigen-4
PD-1	Programmed cell death protein 1
LAG-3	Lymphocyte-activation gene 3
TIM-3	T cell immunoglobulin and mucin domain 3
ORR	Overall response rate
CALR	Calreticulin
ER	Endoplasmic reticulum
SIRPα	Signal regulatory protein alpha
Ig	Immunoglobulin
CR	Complete remission
ADCP	Antibody dependent cellular phagocytosis
TP53	Tumour protein p53
PBMC	Peripheral blood mononuclear cell
OXPHOS	Oxidative phosphorylation
ROS	Reactive oxygen species
FAO	Fatty acid oxidation
HIF-1α	Hypoxia-inducible factor 1-alpha
VEGF	Vascular endothelial growth factor
EGF	Epidermal growth factor
PFKFB3	6-phosphofructo-2-kinase/fructose-2,6-bisphosphatase 3
EZH2	Enhancer of Zeste Homolog 2
HDAC	Histone deacetylases
DNMT3A	DNA methyltransferase 3A
